# Preparatory brain activity and anticipatory postural adjustments accompanied by externally cued weighted-rapid arm rise task in non-specific chronic low back pain patients and healthy subjects

**DOI:** 10.1186/s40064-016-2342-y

**Published:** 2016-05-20

**Authors:** Mehdi Sadeghi, Saeed Talebian, Gholam Reza Olyaei, Behrouz Attarbashi Moghadam

**Affiliations:** Department of Physical Therapy, School of Rehabilitation, Tehran University of Medical Sciences, P. O. Box 113635-1683, Tehran, 1148965141 Iran

**Keywords:** Low back pain, Contingent negative variation, Anticipatory postural adjustments, Electromyography, Cerebral cortex, Preparatory brain activity

## Abstract

**Objective:**

Cortical reorganization is one of the most plausible mechanisms underlying impairment of anticipatory postural adjustments (APAs) in low back pain (LBP) patients. In order to clarify changes in corticomotor neurophysiology, APAs were assessed by using electromyography (EMG) and electroencephalography (EEG).

**Methods:**

An equal number (29) of nonspecific LBP patients and healthy subjects performed unilateral rapid arm movements in response to auditory imperative stimulus preceded by warning stimulus within 2 s interstimulus interval. Burst onset activity was calculated in relation to the activity of anterior deltoid for bilateral transverse abdominis/internal oblique (TrA/IO), and also postural muscles on left side, including rectus abdominis, external oblique (E.O), erector spinae and medial head of gastrocnemius (Gc.M). Contingent negative variation (CNV) potentials were recorded by scalp EEG, and the area under receiver operating characteristic curve (AUC) was analyzed.

**Results:**

In LBP patients, there was a significant onset delay for E.O and bilateral TrA/IO, but a significant earlier activity for Gc.M (for both *P* < 0.05). The CNV parameters were considerably greater in LBP patients (*P* < 0.01). The AUC was significant just for left TrA/IO and E.O muscles (*P* < 0.05).

**Conclusions:**

The CNV amplitudes were increased, and APA onset times re-organized to be delayed at the trunk and early at the distal limb in LBP cases. These findings support the hypothesis about reorganized activity of cerebral cortex in LBP patients.

## Background

Low back pain (LBP) is considered as pain limited to the region between the 12th rib and the inferior gluteal fold, whether it is accompanied by leg pain or not (Krismer and Van Tulder 2007). Based on the scientific literature, there is a high prevalence for LBP which involves 70–85 % of all people during their lifetime so that it is estimated that the mean ± SEM of point prevalence is 11.9 ± 2.0 % and one-month prevalence is 23.2 ± 2.9 % (Andersson 1999; Hoy et al. 2012). Although most LBP cases recover within 4 weeks of pain experience, recurrence of this condition can occur about one year after pain subsidence (Cassidy et al. 2005; Pengel et al. 2003; Wasiak et al. 2006). The pain in approximately 90 % of patients is nonspecific and without any identified organic pathology (Van Middelkoop et al. 2010). LBP imposes a huge burden on society, and its pathophysiology is still unknown in spite of huge investigations in this regard (Langevin and Sherman 2007).

One plausible mechanism that has been mentioned for inducing chronic recurrent LBP is the change in postural control of the trunk muscles (Tsao et al. 2008). Anticipatory postural adjustments (APAs) occurring prior to voluntary movements are necessary for postural control (Mok et al. 2011). LBP patients display delay in the feedforward activation of deep abdominal muscles before onset of focal movement (Hodges and Richardson 1999; Hodges 2001), and also these patients exhibit changes in spatial representation and magnitude of somatosensory-evoked potentials (SEPs) of brain in response to painful or painless stimuli (Flor et al. 1997). Furthermore, alteration of lumbar paraspinal muscle activity occurs in LBP cases due to changes in nervous system including reflex inhibition, muscle’s nerve supply loss and supraspinal changes (Tsao et al. 2011). To explain, there is a loss of discrete control of paraspinal muscles in LBP patients; for instance, superficial and deep multifidus fascicles are recruited simultaneously during trunk perturbation. Cortical reorganization in motor cortex seems to be responsible for the changes in activity of paraspinal muscles (Tsao et al. 2011).

Patients with recurrent LBP have a delay in the feedforward contraction of transverse abdominis (TrA) at the time of performing rapid arm movement task (Hodges 2001; Hodges et al. 2003; Hodges and Richardson 1996, 1999). The delayed onset of TrA contraction is associated with the shifts in motor cortical representation of this muscle (Tsao et al. 2008). Neuroimaging research demonstrated that chronic musculoskeletal pain causes structural and functional cortical reorganization, which may give rise to the evolution and preservation of chronic pain (Wand et al. 2011).

Prior studies suggested that delayed APAs are a potential mechanism of impaired motor control in LBP cases (Hodges 2001; Hodges et al. 2003; Hodges and Richardson 1996, 1997b), and that APAs are pre-programmed by preparatory activity of the two regions of brain, namely premotor and supplementary motor area (Massion 1992); moreover, there is a correlation between delay of APAs and cortical reorganization in LBP patients (Tsao et al. 2008). There are also extensive studies regarding altered postural control strategy in BP patients (Hodges 2001; Jacobs et al. 2009; Mok et al. 2011; Radebold et al. 2000; van Dieen et al. 2003; Henry et al. 2006). Taken together, it seems that changes in supraspinal centers are involved in motor control impairment; therefore, it is important to understand the possible relationship between motor control impairment and changes of corticomotor neurons in LBP patients (Jacobs et al. 2010; Strutton et al. 2003, 2005).

Contingent negative variation (CNV) is a slow negative event-related potential in the brain that can be applied for better understanding of neural mechanisms concerning changes of postural control coordination in painful conditions (Jacobs et al. 2011). The CNV potential includes two defined stimuli: warning (S1) and imperative (S2). During the interval between S1 and S2, there is a negative shift in EEG amplitude, and CNV appears gradually preceding voluntary movement (Tecce 1972; Walter et al. 1964). The late CNV component has been used to measure preparatory brain activity by a number of studies (Brunia 2003; Brunia and van Boxtel 2001; Tecce 1972; Walter et al. 1964), and it contains stimulus anticipation (Brunia and van Boxtel 2001) and postural preparation (Fujiwara et al. 2009; Maeda and Fujiwara 2007). Brain regions involved in CNV generation are prefrontal cortex, supplementary motor and premotor areas (Gemba et al. 1990; Lamarche et al. 1995).

Changes in cortical neurophysiology of voluntary movement have been indirectly related to the three measures of motor control impairments in LBP cases: delay of feedforward postural strategy, decrease in APAs variability and reorganization of cortex (Hodges 2001; Hodges et al. 2003; Hodges and Richardson 1999; Jacobs et al. 2009, 2010; Tsao et al. 2008; Chiou et al. 2014; Strutton et al. 2003; Strutton et al. 2005). Existence of these changes make it necessary to measure directly brain neurophysiological activity involved in altered postural coordination (Jacobs et al. 2010).

However, the anticipatory activity of deep abdominal muscles is not always apparent in healthy subjects performing rapid arm movements, and this may cause uncertainty when it refers to the functional role of feedforward postural adjustments as a protective mechanism against the development of LBP (Allison and Henry 2002; Marshall and Murphy 2003).

Therefore, precise study of the brain activity during voluntary movement is required so as to clarify the mechanisms responsible for APA deficit that has been pre-programmed by central nervous system (CNS) in chronic LBP cases. Accordingly, the present study aimed to measure preparatory brain activity changes in chronic non-specific LBP patients. To the best of our knowledge, this is the first research that evaluates CNV changes during voluntary rapid arm movement in chronic LBP patients.

## Methods

### Participants

Fifty-eight right-handed male individuals participated in our study, which include 29 chronic LBP cases, aged 28.9 ± 5.5 years, from Tehran University of Medical Sciences (TUMS) hospitals and clinics, and the same number of healthy subjects, aged 29.2 ± 5.1 years, matched in control group. Written informed consent approved by the local ethics committees of TUMS was obtained from the participants, and the study procedures conformed to the guidelines of the Declaration of Helsinki.

Participants who had non-specific recurrent LBP with duration of more than 3 months (Van Middelkoop et al. 2010) were included in the LBP group. During the test, LBP participants had least pain, and their symptoms were not exacerbated by experimental procedures. Healthy subjects were participants neither experiencing LBP in the last year nor ever having LBP lasting more than three consecutive days. Subjects were excluded from each group if they had any following problems: known circulatory, neurological, respiratory or vestibular disorders, prior spine or limb fractures, abdominal or spinal surgery, malignancy, systemic infection, hearing impairment, cognitive deficit, apparent postural deformities (i.e. kyphosis and scoliosis) and take any caffeinated food or drink on the day of testing (Maeda and Fujiwara 2007) as well as analgesic, anti-anxiety/sedative or anti-depressant drugs in the past month. On the day of experiment, prior to performing the task, measurements of disability and pain intensity were performed using a Roland Morris Disability Questionnaire (RMDQ) (Mousavi et al. 2006) and 10 cm visual analog scale (VAS), respectively. Table 1 provides demographic data of participants.

**Table 1:** Demographic information of control and LBP subjects

	Control subjects (*n* = 29) mean ± SD	LBP Subjects (*n* = 29) mean ± SD	*P* value
Age (years)	29.2 ± 5.1	28.9 ± 5.5	0.825
Height (m)	1.76 ± 0.06	1.76 ± 0.05	0.685
Weight (kg)	74.2 ± 13.4	77.1 ± 9.7	0.351
Body Mass Index (kg/m^2^)	23.8 ± 3.5	24.7 ± 3.1	0.312
Roland Morris score^a^	N/A	5.5 ± 3	N/A
Visual analog scale^b^	N/A	1.7 ± 0.9	N/A

*P* values are determined by Student’s *t* test. *N/A* not applicable. The level of significance was set at *P* < 0.05

*LBP* low back pain, *m* meter, *kg* kilogram, *kg/m* ^2^ kg/square of meter

^a^Range of Scales is from: 0 to 24

^b^Range of Scales is from: 0 to 10

### Electroencephalography

Slow electroencephalography (EEG) of brain potentials preceding movement was acquired by means of an EEG apparatus (Brain Quick System, Micromed, Italy). Standard EEG was measured using surface silver–silver chloride cup electrodes according to the International 10–20 Electrode Placement System (Klem et al. 1999).

The CNV potentials were recorded from scalp midline channels located at the frontal (Fz), central or vertex (Cz), and parietal (Pz) that referenced to the mastoid protuberance, monopolar EEG recording mode (Khanmohammadi et al. 2015). The participant was grounded by a surface ground electrode placed on the Fpz.

To eliminate the artifacts caused by eye-movements, vertical electro-oculogram (vEOG) and horizontal electro-oculogram (hEOG) were recorded with Ag/AgCl skin electrodes situated above and below the left eye and on the outer canthi of both eyes respectively, in a bipolar montage. Subjects were asked to gaze at a fixed point on the wall, but not blink. Input impedance of both EEG and EOG electrodes was kept below 5 kΩ. The EEG signals were amplified, and band-pass filtered between 0.05 and 60 Hz and sampled at 256 Hz. For further analysis, analog EEG signals were converted to digits by a 32-bit analog/digital (A/D) convertor (Khanmohammadi et al. 2015).

### Electromyography

The focal activity of prime mover and pre-activation of postural muscles were recorded using seven preamplified bipolar active electrodes (*Type NOS.SX230*, *Biometrics Ltd*., *Cwmfelinfach*, *Gwent*, *UK*) with a fixed center-to-center interelectrode distance of 20 mm, recording diameter of 10 mm, built-in differential amplifier with a gain of 1000, input impedance of 10^15^ Ω, common mode rejection ratio of 110 dB at 60 Hz, and bandwidth of 20–450 Hz. A strap ground electrode was fixed around left wrist joint and placed on the radial styloid process. To reduce skin input impedance and improve the quality of electromyography (EMG) signal, careful skin preparation (including hair shaving, skin rubbing with alcohol and abrasion with fine sand paper) was used prior to electrode placement.

The surface electrodes were positioned over right anterior deltoid (AD), unilateral trunk and lower limb muscles on left side, which include rectus abdominis (R.A), external oblique (E.O), erector spinae (E.S), medial head of gastrocnemius (Gc.M) and bilateral TrA/internal oblique (I.O). The electrodes were placed over the bulk of muscles and parallel to muscle fiber orientation.

The locations of electrodes for muscles were as follows: AD, at one finger width distal and anterior to the acromion process over the belly of the muscle (Hermens et al. 2000); R.A, 1 cm above the navel and 2 cm lateral to the midline; E.O, just below the rib cage and along a line connecting the most inferior point of the costal margin and the contralateral pubic tubercle; E.S, 3 cm lateral to the midline at the L3 level (Talebian et al. 2010); Gc.M, on the most prominent bulge of the muscle (Hermens et al. 2000); and TrA/IO, 2 cm inferior and 2 cm medial to anterior superior iliac spine (ASIS) (Marshall and Murphy 2003).

### Procedure

The subjects of both groups were measured while standing without shoes in a relaxed position with their feet shoulder-width apart. In response to an auditory imperative stimulus (S_**2**_) preceded by an auditory warning stimulus (S_1_), each participant performed right rapid unilateral shoulder flexion task from 60° to 90°, concurrently holding a weight equivalent to 3 % of his body weight (Maeda and Fujiwara 2007). Subjects performed the weighted rapid arm flexion; meanwhile, they maintained the elbow joint straight throughout movement.

Maeda and Fujiwara (2007) reported that the magnitude of late CNV component is affected under preparatory periods of <2.0 s and >3.0 s (Maeda and Fujiwara 2007); therefore, the duration of preparatory period or interstimulus interval (ISI) between S_1_ and S_2_ was set 2.0 s (Khanmohammadi et al. 2015). The parameters of auditory stimuli incorporation, intensity, duration and frequency were 60 dB, 100 ms and 2 kHz, respectively (Fujiwara et al. 2009). During the trial, subjects were instructed to keep equal weight bearing and performed weighted rapid arm raise as fast as possible.

**Fig. 1:**
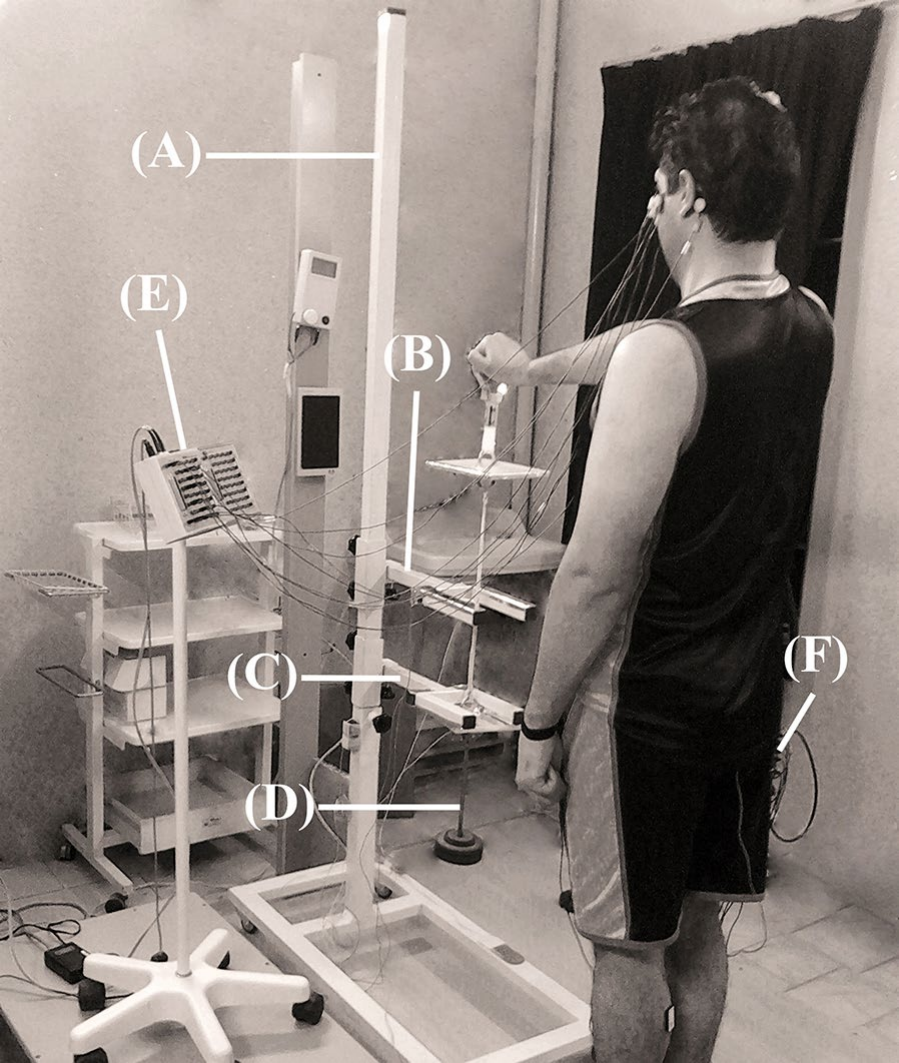
The figure shows Experimental setup was used for weighted rapid arm rise. (*A*) Custom-made Experimental setup. (*B*) The *upper* U-shaped arm adjusts the initial position. (*C*) The *lower* U-shaped arm determines the final position. (*D*) Predetermined load fixed to a handle. (*E*) Electroencephalography (EEG) device. (*F*) Electromyography (EMG) device

Each subject stood in the same position, as previously mentioned in front of custom-made setup and held handle of a predetermined weight while his right upper extremity was in position of initial 60° glenohumeral flexion and outstretch position. From the initial position, in response to movement stimulus, the subjects quickly flexed arm to shoulder height, as final position. For each person, the initial and final positions of arm were determined by adjusting the upper and lower U-shaped arms of setup and the angles measured by a goniometer (Fig. 1).

Background muscle activity was measured before taking task performance. This was important to confirm the consistently of background activity and to facilitate the determination of burst onset activity. If the activity level of any muscles was higher than the rest level, the subject would be instructed to relax the muscles (Hodges et al. 2003; Hodges and Richardson 1997a).

Before the start of experimental trials, five training trials were carried out to familiarize participants with the task. Within a trial block, each subject performed thirty repetitions, and then twenty trials with artifact-free EEGs were selected for analysis. The person who analyzed the data was blinded for this selection. After each five repetitions, subjects had the rest period of 5 min in seated position to prevent fatigue.

### Data analysis

**Fig. 2:**
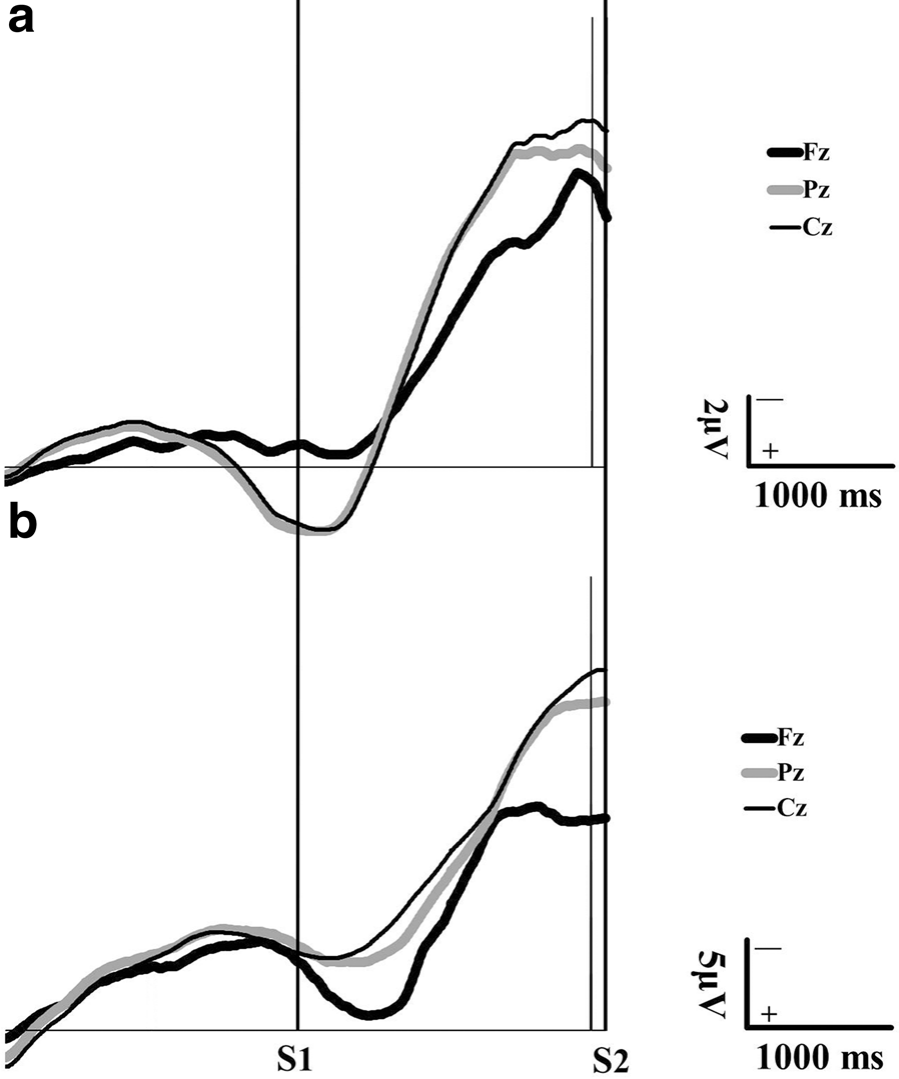
Representative samples of contingent negative variation (CNV) waveforms were recorded from a single subject in each group and electrode location. (*a*) A healthy participant’s average CNV potentials at each electrode location including Fz (*thick black trace*), Pz (thick, gray trace) and Cz (*thin black trace*). (*b*) A LBP patient participant’s average CNV waveforms at each electrode location, which include Fz, Pz and Cz. S1 indicates auditory warning stimulus, and S2 denotes auditory imperative stimulus. The *two thick solid lines* illustrate the interstimulus interval (ISI), and the two spaces between *right tick solid line* and each *thin solid line* represent the 100 ms epoch preceding the imperative stimulus. According to the general agreement, upward deflections in the EEG signal are considered as negative potentials. *Fz* frontal, *Cz* central, *Pz* parietal midline channels, *µV* microvolt, and *ms* millisecond

During the time interval between 500 ms preceding S1 to 500 ms following S2, trials with motion artifacts (voltage at EEG > ± 100 µV) or eye blinks (voltage at EOG > ±100 µV) were excluded for further analysis (Fujiwara et al. 2009; Jacobs et al. 2008; Maeda and Fujiwara 2007; Tomita et al. 2012). The above-mentioned time span has been selected for calculating ensemble-averaged signal in each channel and participant, because for event- related potentials (ERPs), such as CNV, signal averaging reduces noise and enhances signal (Khanmohammadi et al. 2015). The late CNV was measured as the mean of amplitude in ensemble-averaged signal during 100 ms epoch immediately preceding S2, the peak CNV was identified as the maximum amplitude of ensemble-averaged signal from the base line in 1000 ms epoch before S2 (Fujiwara et al. 2011), and the total area under negative deflection of signal between S1 and S2 was calculated for CNV area (Mannarelli et al. 2015) (Fig. 2).

To detect muscle burst onset activity, 40 Hz Butterworth high-pass filter was used to eliminate motion and heart-beat artifacts (Fujiwara et al. 2012). Next, all the filtered raw EMG signals were full-wave rectified, and then smoothed using a moving average of 50 ms time constant (Hashemirad et al. 2009). The AD burst onset activity was determined visually. The onset of each postural muscle was identified when the amplitude of linear enveloped EMG deviated three standard deviations above the mean background EMG activity of the related postural muscle between −300 and −150 ms prior to AD onset latency, and this activity lasted at the minimum of 50 ms (Yaguchi and Fujiwara 2012). The accuracy of computerized algorithm used for onset latency detection was checked by visual inspection.

The onset of postural muscles was considered feedforward if they happened during the time interval from preceding −200 ms to subsequent +50 ms with respect to the onset of EMG activity in the AD muscle (Masse-Alarie et al. 2012). This time interval was selected because the fastest feedback response of postural muscles occurs +50 ms after imposed perturbation (Aruin and Latash 1995; Hodges and Richardson 1997a). AD reaction time was defined as the period between onsets of imperative stimulus and burst activity of AD muscle. The negative value of onset latency indicated earlier activation of postural muscle than AD (Fujiwara et al. 2009).

It is clarified empirically that during rapid arm flexion task, feedforward activity of postural muscles is affected by changes in velocity of arm movement (Jacobs et al. 2010); therefore, maximum angular velocity of the arm movements was measured and checked by two event markers during each trial. In addition to the initial and terminal ranges of arm movement, these event markers showed time span needed for doing the movement task through the entire range. In our pilot study, maximum angular velocity of arm movement was obtained about 170 ± 5 deg s^**−**1^. All the trials with movement velocity less than desired value were eliminated for further analysis.

The responsiveness of dependent variables to discriminate between the two groups, LBP versus healthy control subjects, was examined by the Receiver operating characteristic (ROC) curves analysis. The ROC curve represents true and false positive rates of dependent variables across a series of cutoff points for dichotomous outcome (diseased/non-diseased test results) on Y and X axis, in order. The area under the curve (AUC) is one of the indices of accuracy in ROC curve. The discriminatory ability of the medical diagnostic test is determined by AUC index. The AUC was measured under the range of 0.50 (no accuracy in discrimination between the two groups) to 1.0 (perfect accuracy in differentiation) (Whitney et al. 2005; Strand et al. 2002).

### Statistical analysis

After the results of Kolmogorov–Smirnov (KS) test confirmed the normality of data distribution for all variables, we used the parametric statistical tests for data analysis. Demographic characteristics of subjects were compared between groups with independent *t* test (Table 1). In each group, one-way analysis of variance (ANOVA) for repeated measures was used to determine any significant difference among onset latency of postural muscles, and EEG channels (i.e., Fz, Cz, and Pz) for CNV parameters. In order to compare all the above-mentioned dependent variables between groups (healthy vs. LBP), one-way multivariate analysis of variance (MANOVA) was implemented. Independent *t* test was applied to identify whether there was any difference in AD reaction time between LBP and healthy participants. The effect size was determined by using partial eta-squared value ($\eta_{p}^{2}$). The level of statistical significance (alpha value) was set at 0.05, and SPSS software version 16.0 (SPSS Inc, Chicago, Illinois) used for all statistical analyses. All data were presented as mean ± SD, unless otherwise stated.

## Results

### Demographic characteristics

There were no significant differences in demographic information between participants of the two groups (Table 1); thereby participants of both groups were matched.

### AD reaction time

An independent *t* test was used to determine difference in AD reaction time between healthy and LBP subjects. The null hypothesis of homogeneity of variances was rejected by Levene’s Test [*F* (50) = 10.68, *P* = 0.002]. The result of independent *t* test showed no significant difference between groups [*t* (41.26) = −1.8, *P* > 0.05].

### Onset latency of postural muscles

**Fig. 3:**
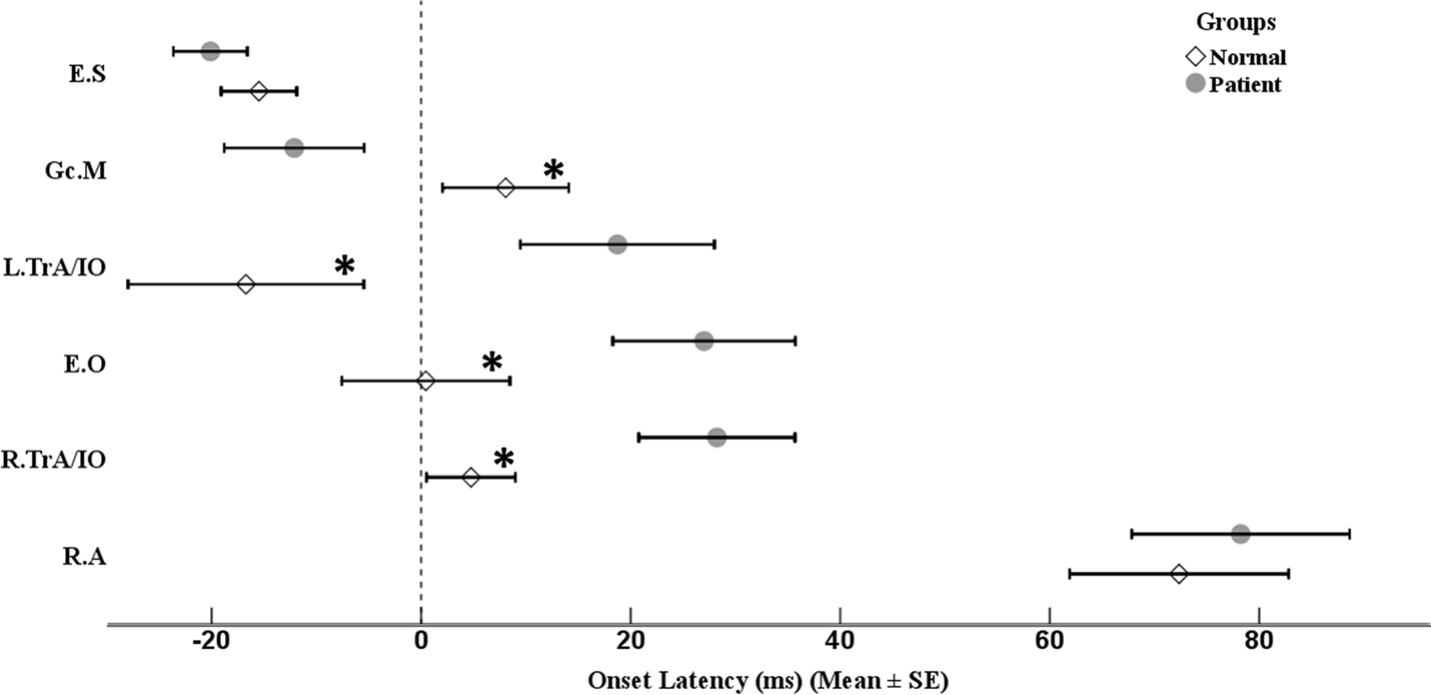
EMG onset of each trunk muscles relative to that of deltoid during rapid arm movement for the control (*empty diamond*) and LBP (*filled circle*) participants. All means are aligned to the onset of deltoid at zero (*dashed line*). *Asterisks* represent significant values (*P* < 0.05). *E.S* erector spine, *Gc.M* medial head of gastrocnemius, *L.TrA/IO and R.TrA/IO* left and right transverse abdominis/internal oblique, *E.O* external oblique, *R.A* rectus abdominis, and *ms* millisecond. Data are expressed as Mean ± SEM

There was a significant main effect of muscle for the healthy [Wilks’ Lambda = 0.171, *F* (5, 24) = 23.33, *P* < 0.0005, $\eta_{p}^{2}$ = 0.83] and the patient group [Wilks’ Lambda = 0. 163, *F* (5, 21) = 21.55, *P* < 0.0005, $\eta_{p}^{2}$ = 0.84]. The results of post hoc comparisons, with Bonferroni corrections, revealed that R.A significantly activated with a delay as compared to other postural muscles in both groups, control (*P* < 0.0005) and LBP (*P* < 0.01). For the healthy subjects, the results of other comparisons showed no significant difference in onset latency (*P* > 0.05), except two comparisons, which include E.S versus R.TrA (*P* < 0.05) and E.S versus Gc.M (*P* = 0.01); nevertheless, for LBP subjects, E.S and Gc.M significantly activated earlier than other postural muscles (*P* < 0.05) (Table 2; Fig. 3).

**Table 2:** The onset latency of postural muscles (mean ± SE) relative to Deltoid burst onset are compared by repeated measure and MANOVA between muscles and groups accordingly

Muscles	Control	LBP	*F* value; groups comparison
R.A	72.34 ± 10.17	78.23 ± 10.74	0.16
E.O	0.45 ± 8.13	27 ± 8.58	5.05*
L.TrA/IO	−16.72 ± 10.16	18.73 ± 10.73	5.76*
R.TrA/IO	4.76 ± 5.74	28.23 ± 6.06	7.91**
E.S	−15.48 ± 3.49	−20.11 ± 3.68	0.83
Gc.M	8.07 ± 6.17	−12.11 ± 6.52	5.06*
*F* value; muscles comparison	19.42***	22.35***	

The negative values indicating burst onset of postural muscles prior to deltoid. Asterisks represent Significant values (*P* value < 0.05)

*LBP* low back pain, *R.A* rectus abdominis, *E.O* external oblique, *L.TrA/IO and R.TrA/IO* left and right transverse abdominis/internal oblique, *E.S* erector spinae, *Gc.M* medial head of gastrocnemius

* *P* < 0.05; ** *P* < 0.01; *** *P* < 0.001

The results of MANOVA test showed a significant main effect of group for onset latency of postural muscles [Wilks’ Lambda = 0 0.743, *F* (6, 48) = 2.77, *P* < 0.05, $\eta_{p}^{2}$ = 0.26]. Post-hoc comparisons demonstrated that the delay in burst onset activity of postural muscles was statistically significant for E.O, L.TrA/IO, R.TrA/IO and Gc.M, but not for E.S and R.A [*F* = 5.05, *P* < 0.05 for the E.O muscle; *F* = 5.76, *P* < 0.05 for the L.TrA/IO muscle; *F* = 7.90, *P* < 0.05 for the R.TrA/IO muscle; *F* = 5.06, *P* < 0.05 for the Gc.M muscle; *F* = 0.84, *P* > 0.05 for the E.S muscle; *F* = 0.16, *P* > 0.05 for the R.A muscle] (Table 2; Fig. 3).

### CNV parameters

**Fig. 4:**
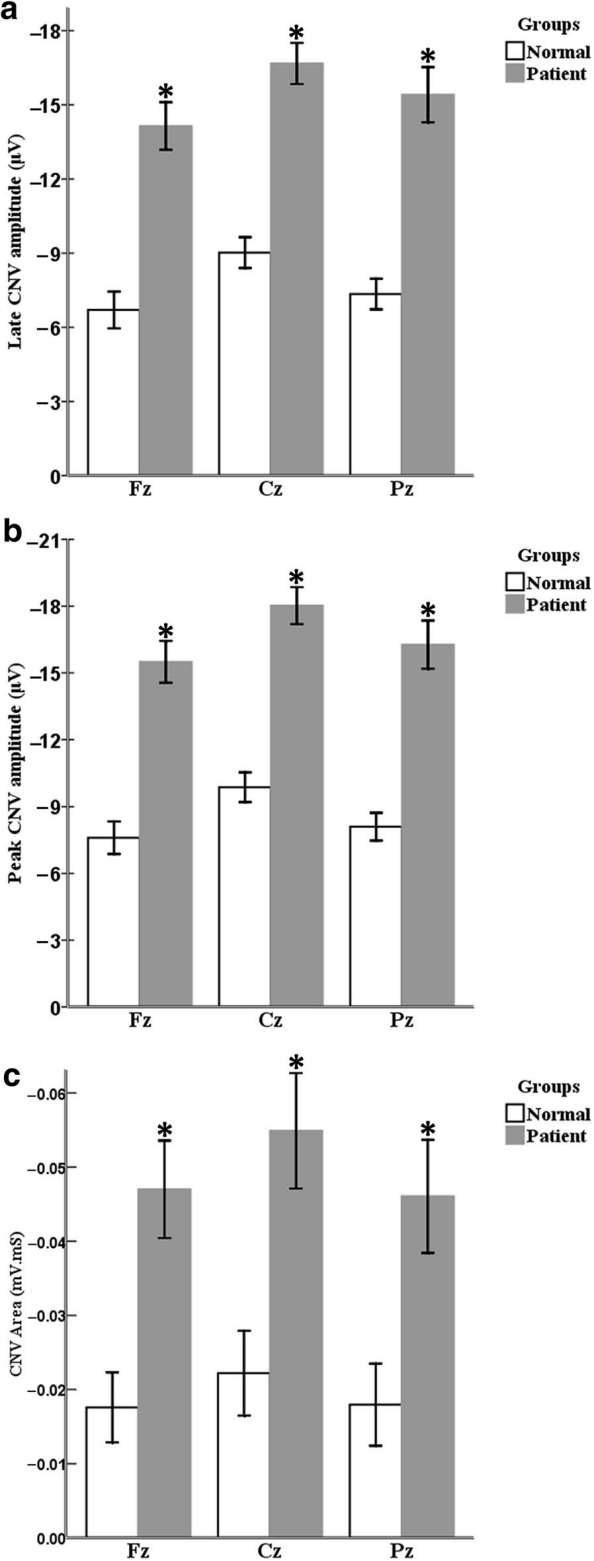
CNV parameters, including (**a**) late CNV, (**b**) peak CNV and (**c**) CNV area of normal and LBP groups, which recorded at each channel separately. Significant values (*P* < 0.05) are represented by asterisks. By convention, negative voltage is plotted upward. *Fz* frontal, *Cz* central, and *Pz* parietal midline channels, *µV* microvolt, and *mV.ms* millivolt.millisecond. Results are represented as mean ± SEM

In the healthy participants, there were significant main effects of channel for the late CNV [Wilks’ Lambda = 0.69, *F* (2, 27) = 6.07, *P* < 0.01, $\eta_{p}^{2}$  = 0.31], peak CNV [Wilks’ Lambda = 0.667, *F* (2, 27) = 6.75, *P* < 0.01, $\eta_{p}^{2}$ = 0.33] and CNV area [Wilks’ Lambda = 0.683, *F* (2, 27) = 6.26, *P* < 0.01, $\eta_{p}^{2}$ = 0.32]. The results of post hoc comparisons, with Bonferroni corrections, demonstrated that late CNV and peak CNV amplitude at Cz channel was significantly greater than Fz and Pz channels (*P* < 0.05). However, no significant difference was between the late CNV amplitude of Fz and Pz channels (*P* > 0.05). The CNV area at Cz channel was significantly greater than Pz channel (*P* < 0.01). There were no significant differences between CNV area of Fz versus Cz as well as Fz versus Pz channels (*P* > 0.05) (Table 3; Fig. 4).

**Table 3:** The repeated measure and MANOVA tests comparing the CNV parameters (mean ± SE) between channels and groups consecutively

CNV parameters	Control	LBP	*F* value; groups comparison
Late CNV (Fz)	6.7 ± 0.86	14.14 ± 0.86	37.36***
Late CNV (Cz)	9.02 ± 0.73	16.67 ± 0.73	54.17***
Late CNV (Pz)	7.35 ± 0.9	15.41 ± 0.9	39.95***
*F* value; Channels comparison	4.35*	1.74	
Peak CNV (Fz)	7.60 ± 0.84	15.49 ± 0.84	43.82***
Peak CNV (Cz)	9.86 ± 0.75	18.02 ± 0.75	58.67***
Peak CNV (Pz)	8.10 ± 0.88	16.27 ± 0.88	42.66***
*F* value; Channels comparison	4.30*	1.79	
CNV area (Fz)	0.018 ± 0.006	0.047 ± 0.006	13.22**
CNV area (Cz)	0.022 ± 0.007	0.055 ± 0.007	11.44**
CNV area (Pz)	0.018 ± 0.007	0.046 ± 0.007	8.89**
*F* value; Channels comparison	1.68	1.56	

Frontal (Fz), Central (Cz), and Parietal (Pz) midline channels. *CNV* contingent negative variation, *LBP* low back pain

Significant values (*P* value < 0.05) are presented by asterisks

* *P* < 0.05; ** *P* < 0.01; *** *P* < 0.001

There was a significant main effect of channel for the peak CNV [Wilks’ Lambda = 0.794, *F* (2, 27) = 3.50, *P* < 0.05, $\eta_{p}^{2}$ = 0.20], but no significant effect for the late CNV [Wilks’ Lambda = 0.80, *F* (2, 27) = 3.32, *P* = 0.051, $\eta_{p}^{2}$ = 0.20] and CNV area [Wilks’ Lambda = 0.865, *F* (2, 27) = 2.11, *P* > 0.05, $\eta_{p}^{2}$ = 0.14] in the LBP subjects (Table 3; Fig. 4).

As compared with control, MANOVA results of the LBP group revealed an increase in late CNV amplitude [Wilks’ Lambda = 0.343, *F* (3, 54) = 34.42, *P* < 0.0005, $\eta_{p}^{2}$ = 0.66], peak CNV [Wilks’ Lambda = 0.308, *F* (3, 54) = 40.37, *P* < 0.0005, $\eta_{p}^{2}$ = 0.69] and CNV area [Wilks’ Lambda = 0.799, *F* (3, 54) = 4.53, *P* < 0.01, $\eta_{p}^{2}$ = 0.20]. Post-hoc comparisons demonstrated that an increase in late CNV amplitude [*F* = 37.36, *P* < 0.0005 for the Fz channel; *F* = 54.17, *P* < 0.0005 for the Cz channel; *F* = 39.95, *P* < 0.0005 for the Pz channel], peak CNV amplitude [*F* = 43.82, *P* < 0.0005 for the Fz channel; *F* = 58.67, *P* < 0.0005 for the Cz channel; *F* = 42.66, *P* < 0.0005 for the Pz channel] and CNV area [F = 13.22, *P* < 0.01 for the Fz channel; *F* = 11.44, *P* < 0.01 for the Cz channel; F = 8.89, *P* < 0.01 for the Pz channel] were statistically significant for all recorded channels (Table 3; Fig. 4).

### ROC curve

**Fig. 5:**
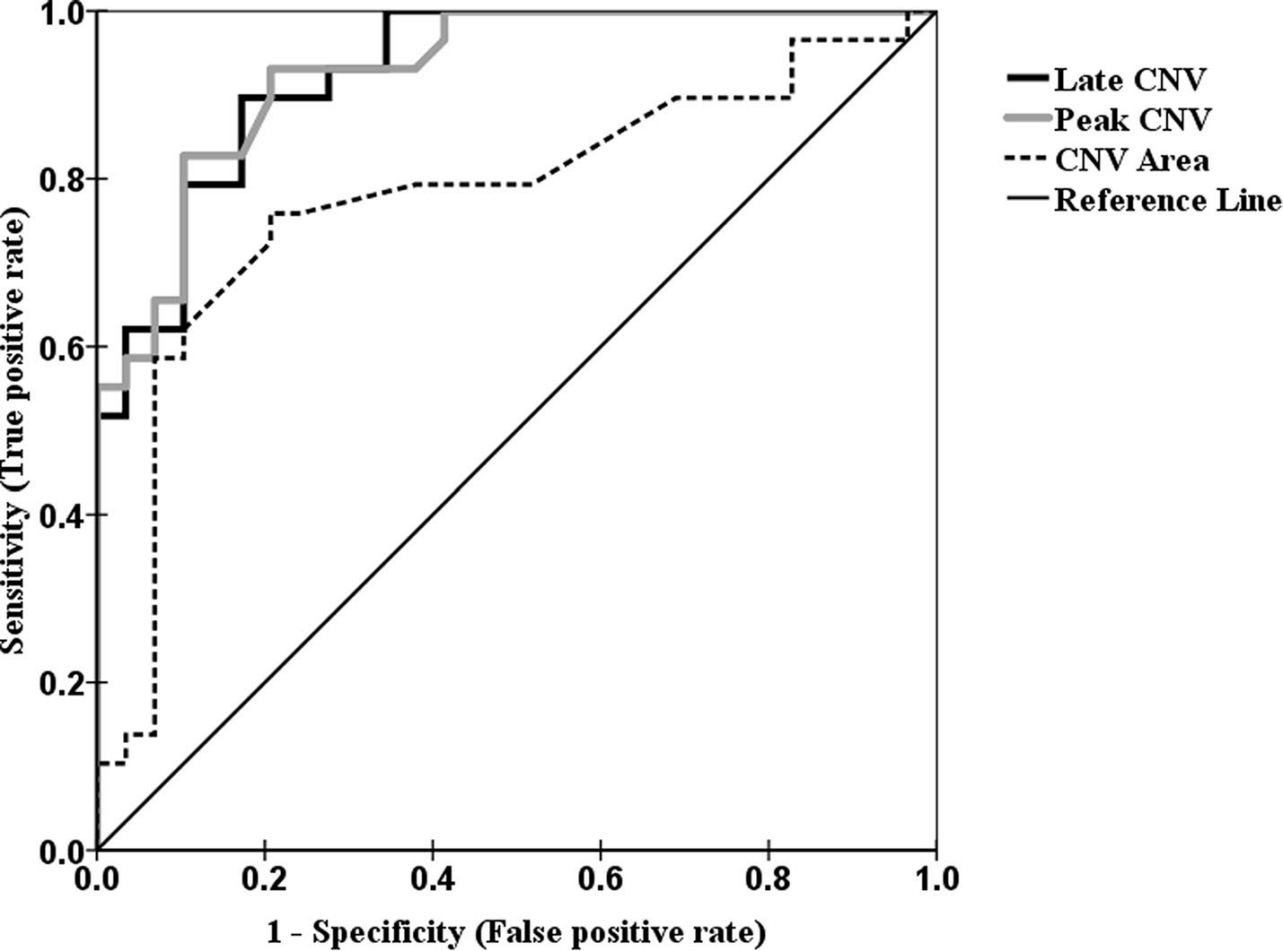
Receiver operating characteristic curve (ROC) of the CNV parameters at Cz channel, comparing discriminative power of the independent variables, including late CNV (*thick dark line*), peak CNV (*thick gray line*) and CNV area (*dashed line*) for detecting participants with LBP versus healthy subjects. The *reference line*, *diagonal of the square*, represents the ability of the diagnostic test in differentiating LBP patients that is no better than chance level (null hypothesis)

Figure 5 illustrates the ROC curve for the discriminative capability of the CNV parameters, which include late and peak CNV and CNV area, at Cz channel to recognize LBP and healthy subjects. The results of the AUC analysis showed a significant curve area [AUC = 0.927, 95 % CI 0.865–0.990, *P* < 0.0005 for late CNV; AUC = 0.928, 95 % CI 0.866–0.990, *P* < 0.0005 for peak CNV; AUC = 0.778, 95 % CI 0.651–0.904, *P* < 0.0005 for CNV area] and rejected the assumption of null hypothesis indicating no discrimination (AUC = 0.50).

The results of the AUC analysis of the ROC curve revealed a significant curve area [AUC = 0.869, 95 % CI 0.778–0.960, *P* < 0.0005 for late CNV (Fz); AUC = 0.893, 95 % CI 0.815–0.971, *P* < 0.0005 for peak CNV (Fz); AUC = 0.758, 95 % CI 0.635–0.881, *P* = 0.001 for CNV area (Fz)] [AUC = 0.850, 95 % CI 0.748–0.952, *P* < 0.0005 for late CNV (Pz); AUC = 0.862, 95 % CI 0.767–0.958, *P* < 0.0005 for peak CNV (Pz); AUC = 0.759, 95 % CI 0.630–0.888, *P* = 0.001 for CNV area (Pz)] in CNV parameters at Fz and Pz channels.

The ROC curve analysis of onset latencies showed significant results [AUC = 0.663, 95 % CI 0.524–0.803, *P* < 0.05 for L.TrA/IO muscle; AUC = 0.655, 95 % CI 0.515–0.796, *P* < 0.05 for E.O muscle] and insignificant results [AUC = 0.503, 95 % CI 0.352–0.654, *P* > 0.05 for R.A muscle; AUC = 0.625, 95 % CI 0.477–0.774, *P* > 0.05 for R. TrA/IO muscle; AUC = 0.584, 95 % CI 0.431–0.736, *P* > 0.05 for E.S muscle; AUC = 0.619, 95 % CI 0.467–0.772, *P* > 0.05 for Gc.M muscle] for area under curves of postural muscles.

## Discussion

The results of this study regarding onset latency of postural muscles revealed that the LBP patients had delayed activation of E.O, L.TrA/IO and R.TrA/IO and earlier recruitment of Gc.M, as compared with the healthy group. The LBP patients also had higher CNV parameters including late CNV, peak CNV and CNV area. The ROC curve findings of independent variables indicated that discriminatory ability of CNV parameters was better than temporal parameters of EMG activity, especially in Cz channel.

The confounding effect of task velocity on the variability of postural muscles burst onset activity is a well-established factor in APAs investigations, and previous studies have reported a consistent postural muscle response upon an arm movement performance above the threshold velocity (Hodges and Richardson 1997b, 1999; Horak et al. 1984). We observed no significant difference in outcomes of AD reaction time between groups indicating no relation between velocity of arm movement and delayed response of postural muscles in the LBP patients.

Consistent with prior investigations, postural trunk muscles of the chronic LBP patients were activated with a delay compared to the healthy subjects (Hodges 2001; Hodges et al. 2003; Hodges and Richardson 1996, 1999). R.A activity was started in a feedback window in the two groups with no considerable difference in muscle onset activity, which is in agreement with other reports (Hodges 2001; Hodges and Richardson 1996).

Generation of reactive forces and displacement of center of mass occur during rapid arm flexion; this displacement causes trunk flexion and the subsequent production of preparatory trunk movements in opposite direction to control the trunk orientation. Preparatory trunk movements lead to sooner rear trunk muscles activation than superficial abdominal muscles in a direction-specific manner to counteract imposed trunk torques due to movement task (Aruin and Latash 1995; Hodges et al. 2000).

The two groups experienced activation of TrA/IO (i.e. two deep abdominal muscles) on both sides of the body in a feedforward manner with a significant difference in activity time in which there was a delayed time of activity in the LBP patients. Past studies in accordance with this work point to the role of anticipatory activation and bilaterally symmetrical contraction of TrA during rapid arm flexion (Hodges 2001; Hodges et al. 2003; Hodges and Richardson 1996, 1999; Masse-Alarie et al. 2012), which was contrary to the findings of Allison et al. (2008) and Morris et al. (2012) who stated an asymmetrical and directional-specific activation of this muscle (Allison et al. 2008; Morris et al. 2012).

Furthermore, Masse-Alarie et al. (2012) have reported that TrA is activated in an asynchronized manner in the LBP patients (Masse-Alarie et al. 2012) may be due to performing free-load rapid arm flexion. In the current study, however, the participants carried out weighted rapid arm flexion; weight parameter imposes greater inertia and reactive forces to the trunk, thereby CNS has to pre-programme more intense postural adjustment mechanisms so as to maintain a balance and center of gravity within the base of support. Our findings exhibited that E.S, but not TrA which was reported by prior investigations (Hodges 2001; Hodges et al. 2003; Hodges and Richardson 1996, 1999), was the first muscle recruited in the healthy subjects; using different EMG recording techniques and different movement task for experimental protocol (surface EMG from TrA/IO in our study vs. fine wire EMG from TrA in other works) is the presumable explanation for the existing controversy.

Mok et al. (2004) documented that the LBP patients have greater activities of lumbopelvic region muscles, especially E.S, leading to decreased lumbar spine and hip motion and the subsequent reduction of hip strategy implementation for balance control. The present study showed that Gc.M had significant earlier activation in the LBP patients than the healthy subjects, while E.S onset latency showed no considerable change. We speculate that the LBP patients prefer to use ankle strategy against postural disturbance produced by weighted rapid arm flexion; this is why there was an earlier activation of Gc.M in these cases than the healthy subjects.

In our study we demonstrated, for the first time, that late CNV amplitude during rapid arm flexion significantly increased in the LBP patients than the healthy participants. CNV is a slow cortical negative potential and an index of anticipatory behavior. Late CNV is the sum of two components, namely movement-preceding negativity (MPN) and stimulus-preceding negativity (SPN), unlike other movement-related cortical potentials (MRCPs) [e.g., Bereitschaftspotential (BP)]. MPN component indicates motor preparation for the movement execution, and SPN component shows an anticipatory attention for S2 (Brunia 2003). There is a direct and positive relationship between the CNV magnitude and extent of attentional resources allocated to accomplish the task, level of muscular effort needed to perform the movement, speed of movement and experimentally-induced pain (Jacobs et al. 2011; Brunia 2003; Stude et al. 2003).

LBP patients prefer to do simultaneous co-contraction of postural muscles rather than using anticipatory postural mechanisms during postural perturbations. Nociception and damage of supporting tissues lead to proprioceptive acuity reduction, and these patients implement a robust strategy by increasing spine stiffness to prevent spinal instability. Studies have demonstrated that LBP patients have the higher prefrontal cortex activation in postural control, suggesting that they have more cognitive spinal control than the healthy subjects (Van Dieën and Kingma 2013; Jacobs et al. 2010; Wand et al. 2011).

Prefrontal cortex is one of the generator sources of CNV (Maeda and Fujiwara 2007), and that amplitude of CNV is monotonically and positively associated with attention (Tecce 1972). More involvement of prefrontal cortex and the resulting postural control cognition is the reasonable explanation for higher CNV amplitude induction in the LBP patients than the healthy subjects of the present study. It is pertinent to point out the existence of a compensatory mechanism and involvement of cognitive centers to withstand imposed external perturbations in the LBP patients; in other words, postural task is more difficult and challenging in these cases than the healthy subjects.

There is a growing interest in recent studies toward understanding of upstream changes in motor control neurophysiology of LBP cases (Chiou et al. 2014, 2015; Jacobs et al. 2010, 2011; Strutton et al. 2003, 2005; Tsao et al. 2008, 2011). Jacobs et al. (2010) reported that LBP patients have changes in neurophysiology of cortex motor area during self-initiated movements (Jacobs et al. 2010). Our results showed simultaneous changes of CNV, as the neurophysiologic index of brain function, and the delay in postural muscles in the LBP patients, which was in agreement with previous results (Chiou et al. 2014, 2015; Jacobs et al. 2010, 2011; Strutton et al. 2003, 2005; Tsao et al. 2008, 2011). However, there was no significant correlation between CNV changes and altered trunk muscle onset latency, and altered CNV and impaired APA are probably two independent but coexistent phenomena.

Furthermore, the LBP group had no significant change in CNV amplitude between channels, while healthy subjects of this study had a maximum CNV amplitude at vertex (so called Cz) channel, as reported by others (Low and McSherry 1968; Ruchkin et al. 1986; Brunia 2003; Tecce 1972), probably due to the area of Cz channel positioning in close proximity to the primary motor cortex (M1) portions responsible for the somatotopic representation of body parts to control posture at the time of performing movement task (Jacobs et al. 2011). Previous study provides evidence that transcranial magnetic stimulation (TMS) application immediately preceding APA onset induces motor evoked potentials (MEPs) of a muscle involved in APA rather than a muscle involved in voluntary movement (MacKinnon et al. 2007).

Based on the results of the AUC analysis of various EEG and EMG measurements, the ability to recognize diseased and non-diseased participants was more discriminative in CNV parameters than temporal parameters of EMG; it is also worth pointing out that CNV channels (Fz, Cz and Pz) and dependent variables (late CNV, peak CNV and CNV area) comparisons showed the most predictive capability for Cz channel and peak CNV but the least predictive ability for Pz channel and CNV area.

Since in this study diagnostic ability for postural muscles onset latency was maximum in L. TrA/IO but minimum in R.A, it is likely that the R.A discriminatory ability was no better that tossing a coin to distinguish diseased cases from non-diseased participants, thus it seems that the peak CNV at Cz channel is a diagnostic test with high accuracy in LBP patients.

## Conclusion

Our results offer novel insights into potential occurrence of anticipatory postural impairment, but without any significant correlation, with CNV changes in LBP patients. In spite of much research in this regard, there are ambiguities in the pathophysiology of LBP and effectiveness of the proposed therapeutic interventions. Therefore, it would be helpful to have a better understanding of central changes in LBP in order to develop new rehabilitation programs based on recovery of motor control impairments, suggesting the necessity of further investigations to elucidate neurophysiological corticomotor changes of LBP.

## Limitations of the study

The major limitation of the present study was that only male subjects participated in the trials and the effect of gender on dependent variables was not measured.

## Abbreviations

APAs: anticipatory postural adjustments
NSCLBP: non-specific chronic low back pain
EMG: electromyography
EEG: electroencephalography
AD: anterior deltoid
TrA/IO: transverse abdominis/internal oblique
R.A: rectus abdominis
E.O: external oblique
E.S: erector spinae
Gc.M: medial head of gastrocnemius
CNV: contingent negative variation
AUC: area under receiver operating characteristic curve
SEPs: somatosensory-evoked potentials
S1: warning stimulus
S2: imperative stimulus
TUMS: Tehran University of Medical Sciences
RMDQ: Roland Morris Disability Questionnaire
VAS: visual analog scale
Fz: frontal
Cz: vertex
Pz: parietal
vEOG: vertical electro-oculogram
hEOG: horizontal electro-oculogram
A/D: analog/digital
ASIS: anterior superior iliac spine
ISI: interstimulus interval
ERPs: event-related potentials
ROC: receiver operating characteristic
KS: Kolmogorov–Smirnov
MANOVA: multivariate analysis of variance
$\eta_{p}^{2}$: Eta-squared value
CNS: central nervous system
MRCPs: movement-related cortical potentials
BP: bereitschaftspotential
MPN: movement-preceding negativity
SPN: stimulus-preceding negativity
M1: primary motor cortex
TMS: transcranial magnetic stimulation
MEPs: motor evoked potentials
